# Recent Progress on Passive, Thermally Localized Solar-Driven Multistage Water Evaporation

**DOI:** 10.3390/membranes13050460

**Published:** 2023-04-24

**Authors:** Hyeon Tae Kim, Morteza Afsari, Noel Peter B. Tan, Ho Kyong Shon, Leonard D. Tijing

**Affiliations:** 1 Faculty of Engineering and Information Technology, University of Technology Sydney, P.O. Box 123, 15 Broadway, Ultimo, NSW 2007, Australia; hyeon.t.kim@student.uts.edu.au; 2 Centre for Technology in Water and Wastewater, School of Civil and Environmental Engineering, University of Technology Sydney, P.O. Box 123, 15 Broadway, Ultimo, NSW 2007, Australia; morteza.afsari@student.uts.edu.au (M.A.); hokyong.shon-1@uts.edu.au (H.K.S.); 3 Department of Chemical Engineering, College of Technology, University of San Agustin, Iloilo City 5000, Philippines; dtan@usa.edu.ph; 4 Center for Advanced New Materials, Engineering, and Emerging Technologies (CANMEET), University of San Agustin, Iloilo City 5000, Philippines; 5 ARC Research Hub for Nutrients in a Circular Economy, University of Technology Sydney, P.O. Box 123, 15 Broadway, Ultimo, NSW 2007, Australia

**Keywords:** thermally localized, solar water evaporation, passive desalination, multistage distillation, water purification, solar energy, evaporator, absorber, membrane, photothermal material

## Abstract

Thermally localized solar-driven water evaporation (SWE) in recent years has increasingly been developed due to the potential of cost-efficient freshwater production from small-scale portable devices. In particular, the multistage SWE has attracted much attention as the systems possess mostly a simple foundational structure and high solar-to-thermal conversion output rates, enough to produce freshwater from 1.5 L m^−2^h^−1^ (LMH) to 6 LMH. In this study, the currently designed multistage SWE devices were reviewed and examined based on their unique characteristics as well as their performances in freshwater production. The main distinguishing factors in these systems were the condenser staging design and the spectrally selective absorbers either in a form of high solar absorbing material, photovoltaic (PV) cells for water and electricity co-production, and coupling of absorber and solar concentrator. Other elements of the devices involved differences such as the direction of water flow, the number of layers constructed, and the materials used for each layer of the system. The key factors to consider for these systems include the heat and mass transport in the device, solar-to-vapor conversion efficiency, gain output ratio (representing how many times the latent heat has been reused), water production rate/number of stages, and kWh/number of stages. It was evident that most of the studied devices involved slightly different mechanisms and material compositions to draw out higher efficiency rates from the current limitations. The reviewed designs showed the ability to be adopted into small-scale solar desalination allowing for accessibility of sufficient freshwater in needing regions.

## 1. Introduction

Water scarcity is regarded as a significant global challenge, with approximately one-third of the world’s population living in water-stressed countries [1]. Water desalination has been one of the main contributors in addressing the increasing demands and depletion of fresh water. However, there have been adverse issues in saltwater desalination as they require high levels of energy for water production, maintenance, and clean-up of salty waste [2,3]. There is an urgency to allow for the affordability of desalination technologies for low-income and lower-middle-income countries [4,5]. The current desalination technologies mostly used are based on reverse osmosis (RO) processes (state of the art) and thermal desalination. However, they are energy-intensive, mostly provided from pollutive non-renewable sources, and expensive in construction and operation [6]. Solar thermal desalination technologies have the potential to provide modular, low cost and efficient systems that address the issues outlined above. Moreover, SWE desalination has evolved in recent years due to advancements in thermal localization and its applications for passive water desalination allowing for possible resolutions in regions of freshwater shortages with limited or no water and energy infrastructures [7]. The single-stage SWE devices have allowed for the initial development of modular SWE desalination devices with affordable costs [8]. However, it has faced challenges in its heat and mass transport due to its theoretical limits, and thus, only providing an efficiency level that conventionally cannot exceed 100% as latent heat is lost through vapor condensation into the environment [9]. As these devices are being developed for localized use in households and smaller communities, the production of water is very low compared to large desalination plants.

Portable multistage SWE desalination provides an opportunity to resolve some of these issues by system re-configuration wherein the supposedly lost latent heat for condensation is now being reused. According to the World Health Organization (WHO), 2 L of safe water is necessary for basic consumption and 7.5 L for lactating women undertaking moderate physical activity. The current multistage SWE desalination devices being developed have been able to produce freshwater almost satisfactory to the basic requirements for daily water consumption; however, several design and operational challenges are still needed to be addressed for its practical implementation. Recent development stage for multistage SWE devices reported in the literature [9,10] have managed to reach a production rate of above 5 L m^−2^h^−1^ (LMH). These results are enough to provide daily safe drinking water for small households. Thus, there is an increasing interest in multistage SWE to have practical application for freshwater production in inland and remote communities compared to single-stage interfacial SWE. Although there have been several review articles on SWE, most of them reported mainly on single-stage SWE and their designs and challenges [11,12,13,14], and touched sparsely on multistage SWE, which may have more practical significance for public use. Thus, this review focuses on the recent updates and progress of passive multistage portable SWE desalination systems which are still in their development and optimization stages. The review includes an analysis of the latest development stage systems and compares the design and operation of each device to determine its design and possible economic limitations. Through this, identification of quantitative strengths and weaknesses has been outlined for improvements in efficiency and performance.

## 2. Overview of Passive Solar Water Evaporation (SWE) 

### 2.1. Single-Stage SWE

SWE has gained much interest in the past decade due to its potential sustainable approach for freshwater production at a household and community level, especially for those lacking access to clean water and energy. Aside from the development of new photothermal materials and designing of water pathways [15,16,17], the design of the evaporation layer configuration is one of the most important key design parameters to improve the overall performance. The conventional SWE is configured into front-side or back-side evaporators. The former involves an interfacial heating design where solar absorbers are usually floated on top of the feed water and evaporation takes place on top of the evaporator. The latter is the opposite wherein a photothermal material or a PV cell is used as the heating material, and the evaporation takes place at the bottom side of the evaporator and is likewise condensed. 

Both methods operate fundamentally differently and involve various steps and layers for overall operation. Front-side single stage SWEs require the front layer to be multifunctioning as a solar absorber and an evaporator as the layer is located above the feed water. The condensing layer material requires the solar flux to reach the solar absorber. This significantly reduces the types of materials which can be used in this mechanism. To draw high levels of efficiencies, the materials for the solar absorber and evaporator can be composed of carbonaceous materials, plasmonic structures, and polymeric materials [18]. When considering performance as a factor, these materials can be costly and complex to develop, manufacture, and practically implement. Back-side single stage SWEs can have layers separated in single functioning materials due to the direction of condensation. This allows for a greater range of materials to be chosen from, including cost-effective and high-performing materials widely available. Although the mechanism will require a slightly more complex operating system, the separation of layers allows for more potential to draw out performance and utilize latent heat recovery. Multistage variations of SWE have been widely researched using this mechanism due to more options for adjustments to design and creative methods for higher solar-to-vapor conversion rates. 

### 2.2. Multistage SWE

**Figure 1:**
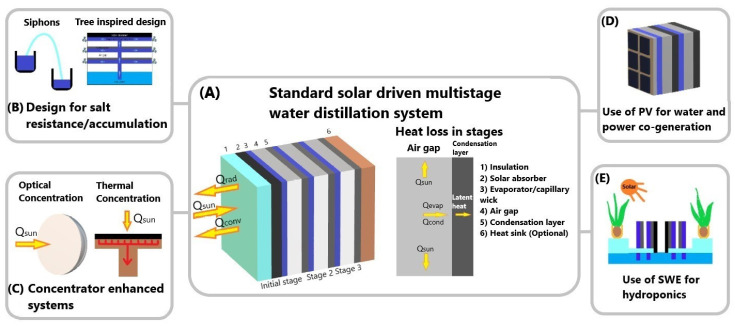
Various designs of multistage water distillation systems reported in the literature: (**A**) Standard solar-driven multistage distillation system, (**B**) Design for salt resistance/accumulation, (**C**) Concentrator enhanced systems, (**D**) PV for co-generation of electricity and water (**E**) Hydroponic systems.

In conventional single-stage SWE design, much of the latent heat used for condensation is lost to the ambient environment. Re-utilizing of this wasted latent heat to further use for water evaporation is a good approach to increase the overall efficiency, and thus, multistage SWE devices have been developed. The technical mechanism of the passive portable multistage SWE device is designed under the universal desalination process similar to single-stage SWE but with multiple condensers. It consists of a solar absorber, a capillary wick/evaporation chamber, an air gap, and a condensation layer. However, in the process of heat and mass transfer for a passive solar desalination process, there are other areas where heat is lost due to the nature of the design. Thus, achieving 100% efficiency is theoretically not possible, resulting in the upper echelon efficiency of approximately 70%. The energy loss from convection and radiation is a matter of material performance which is an existing issue to resolve in multistage SWE desalination and needs to be addressed based on a combination of different factors such as cost, environmental sustainability, etc. Figure 1 illustrates the multistage device where the latent heat is utilized for increasing efficiency. Although the layout and the mechanism of these devices follow a similar pattern, the detailed designs alter in different prototypes such those optimizing the absorber and stage design, the use of solar concentrator and photovoltaic (PV) cells, and other designs for salt mitigation or recovery. 

From Figure 1A, the first stage of SWE design is the solar absorber, where the solar flux is absorbed and converted to heat energy to heat the saltwater absorbed in the capillary wick. Spectrally selective absorbers (SSA) are most used in this layer as they possess high solar radiation absorptance and low thermal radiation emittance, thus achieving an efficient solar thermal conversion [19]. However, alternatives such as black paint or other high solar absorbing materials are used in this layer for a reduction in costs and availability. The total solar flux to heat conversion is utilized by the capillary wick or evaporation layer to produce vapor to be collected in the condensation plate. The vapor production process is repeated after these steps depending on the total number of stages in the system. The number of stages relative to the gain output ratio (GOR) is critical to the efficiency of the device and is examined above. 

The theoretical and practical limitations of single-stage desalination systems are prominent, and thus, utilization of latent heat loss is a viable method to address the restraint in solar-to-vapor conversion efficiency. This is the main indicator for the overall efficiency of the system, which involves the process of heat and mass transfer through solar heat localization and is represented as,

$$\eta =\frac{\dot{m}h_{fg}}{q_{solar}^{\prime \prime }A}$$

where η is the solar evaporation efficiency, $\dot{m}$ is evaporation rate, $h_{fg}$ is the latent heat of vaporization, $q_{solar}^{\prime \prime }$ is the incident solar flux, and *A* is the effective absorber area. 

The solar-to-vapor conversion efficiency is an adequate representation for freshwater production in multistage systems as the recyclable latent heat can be determined. Furthermore, the device’s ability to recycle and retain the outgoing heat within the system in each following stage improves the overall efficiency and water production. The key aspect to consider is the materials used as they alter the performance of the device according to its surface vapor temperature, evaporation rate, thermal efficiency, and salinity in relation to the applied solar concentration [20]. Moreover, the air gap thickness applied to the multistage system significantly impacts the solar-to-vapor conversion efficiency [18].

**Figure 2:**
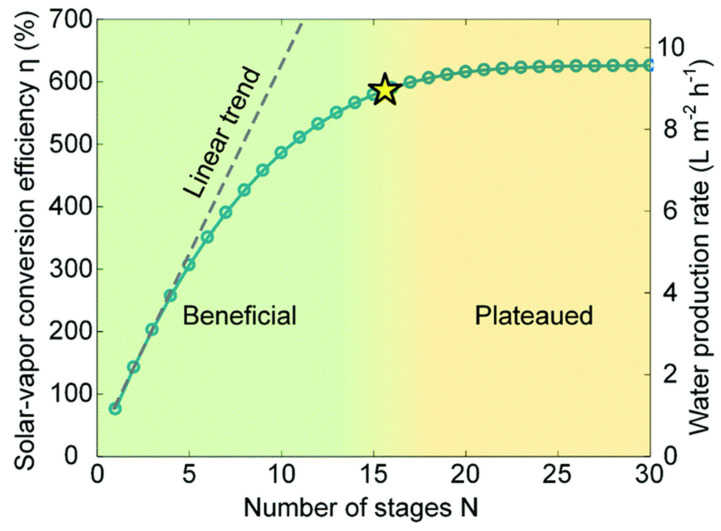
Comparison between number of stages in a multistage SWE vs. performance. The star refers to the transition point where increasing number of stages becomes negligible effect on water production. (Adapted from [18]).

In multistage SWE devices, the GOR can exceed 100% due to latent heat recycling if the system in its first stage is able to retain much of the Q_sun_ (solar flux). The heat recycling process post-heat retention in the initial stage allows for the multistage device to output solar-to-vapor conversion efficiency above 100%. Thus, the performance is relative to the net value of solar-to-vapor conversion efficiency of the initial stage. Given the performance of the system is satisfactory for recycling latent heat, the limitation of the number of layers is bounded by the device’s ability to retain the heat from sidewall heat loss throughout the system. As the ambient temperature must be below the Q_out_ of each layer for condensation to occur, where the minimization of heat loss through sidewalls is crucial. The relative increase in solar-to-vapor efficiency according to the number of layers has been analyzed by Zhang et al. [18], and theoretically modeled to be at approximately 600% (GOR > 6) (see Figure 2). The GOR for multistage SWE devices demonstrated a linear increase to the fifth stage; it then plateaued under 600% at approximately the fifteenth stage due to the side wall heat loss accumulated from each condenser stage [18].

In addition to thermal insulation, the heat and mass transport throughout the system significantly impacts the overall performance of multistage SWE devices. One study [18] has demonstrated an optimal air gap between the evaporator and condenser to be 0.3 cm, with incremental increases resulting in a total net heat loss in the system. Another study [10] utilized a hydrophobic membrane that prevented salt contamination in the freshwater condensation as it acted as a physical barrier. The performance difference in heat and mass transfer between a hydrophobic layer and air gap was negligible with a slight cost increase when it was utilized.

## 3. Applications and Configurations of Thermally Localized Passive Multistage SWE

Various groups have reported on the design and performance of different multistage SWEs for different applications—such as desalination, resource recovery, and water purification—with varying results. When compared with single-stage SWE, the multistage SWE generally has higher water production rate and improved efficiency. However, the optimized design of the solar absorber, number of stages, heat sink, and resource recovery option still faces many challenges. In this section, the latest developments in multistage SWE device design and their performances are discussed. Table 1 provides a summary of different multistage SWEs reported in the literature and their comparison.

### 3.1. Solar Absorber Design and Distillation Stage Optimization for Desalination and Water Purification 

**Figure 3:**
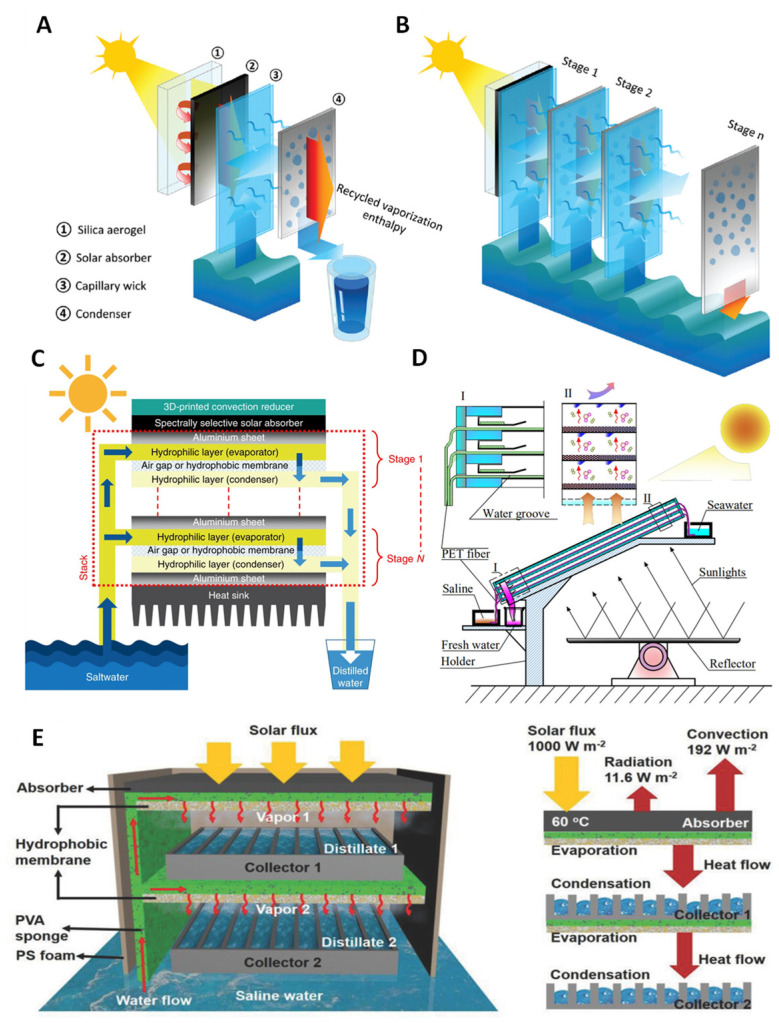
Various designs of multistage SWE: (**A**,**B**) TMSS desalination device [9], (**C**) passive multistage solar desalination device [10], (**D**) multistage solar distiller [21], and (**E**) solar thermal MD system [22].

Direct solar desalination processes have low efficiency as bulk water heating is needed and the seawater itself has poor solar absorption. Furthermore, there is a large amount of heat loss due to its system configuration, making its application less practical. The use of photothermal materials and other solar absorbing materials as interfacial surface configurations have vastly improved the overall system process efficiency. The multistage SWE also mainly uses photothermal materials as the main solar absorbers that induce evaporation to produce clean water. Several groups have tried to improve the efficiency of solar-to-heat conversion by designing new absorber designs from 2D designs to 3D shapes and optimizing the number of distillation stages (see Figure 3). 

Xu et al. [9] designed and tested a thermally localized multistage solar still (TMSS) which achieved very high freshwater production under a passive system (Figure 3A,B). It has achieved a solar-to-vapor conversion efficiency of 385%, producing around 5.78 LMH of clean water under 1 solar illumination [9]. The device consists of 10 stages, which have achieved a GOR larger than 4, demonstrating the efficient and sufficient use of latent heat in the system. The device follows the standard flow process of a multistage SWE device with 4 layers consisting of an insulating layer, solar absorber, capillary wick, and condenser. The material composition of the device is a transparent silica aerogel which is utilized as a thermal insulator and restricts heat loss from conduction, radiation, and convection due to its low thermal conductivity. The solar absorber is a commercially available SSA that is covered with anti-reflective glass. TMSS uses a layer of cheap paper towels as a capillary wick to keep the cost of the device low and maintain adequate wicking performance without causing a bottleneck in the system. However, the wicking ability of cellulose fiber materials significantly decreases as the overall size of the device increases. This imposes a challenge to solve when applied practically. To prevent this, it has been suggested that producing multiple 10 cm × 10 cm devices over a specified area may be sufficient for practical water production. The condensation layer is a 0.5 mm aluminum plate with a hydrophobic coating (Teflon AF), which is separated from the capillary wick by a 5 mm air gap. The construction of the device is put together by a 3D-printed nylon frame for thermal insulation benefits. Approximately 70% of the cost for the material composition is from the 3D-printed nylon frames where the overall price of the device can be reduced. Although TMSS has achieved the highest efficiency as a passive SWE desalination device, bottlenecks are still present in areas such as device geometry, number of stages, and sidewall thermal insulation. Moreover, there has been limited or no application to social and environmental contexts as considerations for diverse weather and social conditions. Thus, the development still delves into the possibility of the application of the device. 

Chiavazzo et al. [10] have examined the potential of multistage SWE desalination devices by comparing the distilled water production relative to the solar energy input into the system. Moreover, they also compared SWE and membrane distillation (MD) systems, where a hydrophobic membrane is implemented between evaporation and condensation. The design and development of the device are based on a passive system with no mechanical systems and utilized solar absorption under one sun (Figure 3C). They have designed a 10-stage system that has achieved a high-water production rate in a laboratory test of almost 3 L m^−2^h^−1^. The initial stage of the device uses a commercially available SSA which is insulated by three 2-mm transparent low linear low-density polyethylene (LLDPE), attached to the multistage system with each layer consisting of 2 hydrophilic layers separated by an air gap or a polytetrafluorethylene (PTFE) microporous hydrophobic membrane. A thermally conductive aluminum plate covers the top of each stage to reduce heat loss and acts as a heating mechanism for the evaporating hydrophilic layer. The inclusion of either an air gap or PTFE microporous hydrophobic layer is considered in this study as the main difference in performance is concerning the possibility of brine contamination in the condensation and thickness of the air gap. The device has an aluminum heat sink for dissipation of residue latent heat into the atmosphere which can be improved.

Ma et al. [21] have developed a 3-stage multistage solar-stiller focusing on brine discharge (Figure 3D). Although the research was not mainly focused on the water production output efficiency, the device was able to reach a GOR of 196% and water productivity of 11.99 kg d^−1^m^−2^ over a 9 h period, which converts to roughly 1.33 kg m^−2^h^−1^. There is no initial insulating layer for this system above the solar absorbing layer. The construction of the device is in a similar layout to the TMSS with different materials used for each of the functioning layers. The solar absorbing layer is an aluminum plate with a black matt polyurethane paint coating which acts as an SSA. The capillary wick utilizes improved polyester fiber (PET fiber), possessing super hydrophilic properties separated by a 9.35 mm air gap from the aluminum condensing layer. The wall is constructed with 4 mm acrylic plates for thermal insulation and no heat sink is attached. Aside from the limitations of the siphoning mechanism, the main challenge faced is the improvements to heat transfer. This is directly correlated to the efficiency and performance of the device, as currently, the solar absorbance is through the bottom of the system. This is inconvenient for practical uses; however, it is useful for the mass transfer of vapor due to buoyancy. Xue et al. [22] conducted a study on a 2-stage device and achieved freshwater production of 1.02 kg m^−2^h^−1^ under 1 sun. The system follows a unique multistage configuration and includes a thermal membrane composed of poly (vinylidene fluoride-co-hexafluoropropylene) (PVDF-HFP) nanofibers in the evaporating chamber (Figure 3E). Unlike other standard designs, the vapor is collected within the evaporation chamber using a collector. The SSA used is a commercially available cermet-coated aluminum alloy substrate without an insulating layer. Outdoor experiments in Wuhan on 26 July 2017, between 7:45 am and 4:45 pm displayed enough water production for an adult usage per day of 3.67 kg m^−2^h^−1^. 

A recent study has taken a new approach in optimizing the design of solar absorbers. Li et al. [23] have examined the development of a solar driven desalination system, from the possible performance improvements by adopting 3D evaporators with increased evaporation to absorption surface ratio. Thus, increasing the evaporation surface area relative to the solar absorption surface area can improve the evaporation rate due to the “photothermal conversion energy allocated to the latent heat of vapor”. However, this is limited due to the high latent heat vaporization of water, where employing a multistage system significantly improves the performance. The device involves a 10-stage passive multistage device (MSD) constructed of SSA insulated by a layer of ethylene-vinyl acetate copolymer (EVA), covered by an anti-reflecting glass. The use of EVA in place of an airgap is unique in its design as it assists in further reducing heat loss. Wool pulp paper was used as the wicking material and copper foil was used for the condensation layer and latent heat recovery. A layer of EVA foam was attached to the back of each layer to act as an evaporation chamber and was also used in conjunction with a heat sink for final heat dissipation. The experimental phase involved testing 2 different single-stage MSD devices, TB-MSD and TD5-MSD, where the evaporation areas were 16 cm^2^, 48 cm^2^, and 80 cm^2^. The solar absorption area was kept constant at 16 cm^2^ to compare the difference in the performance of the evaporation to absorption surface ratio. TB-MSD and TD5-MSD produced evaporation rates of 0.86 kg m^−2^h^−1^ and 1.19 kg m^−2^h^−1^. The TD3-MSD was included in calculations for latent heat utilization where the ribbed evaporation layers (ELs) showed higher average latent heat utilization ratio. However, EL-1 and EL-5 produced better “evaporation and latent heat utilization performance” than EL3, “suggesting the higher thermal dilution ratio” was beneficial for freshwater production. These results determined the theoretical evaporation rate limit of utilizing the ribbed EL was approximately 4.4 kg m^−2^h^−1^.

### 3.2. Concentrator-Enhanced Multistage Solar Water Evaporator 

**Figure 4:**
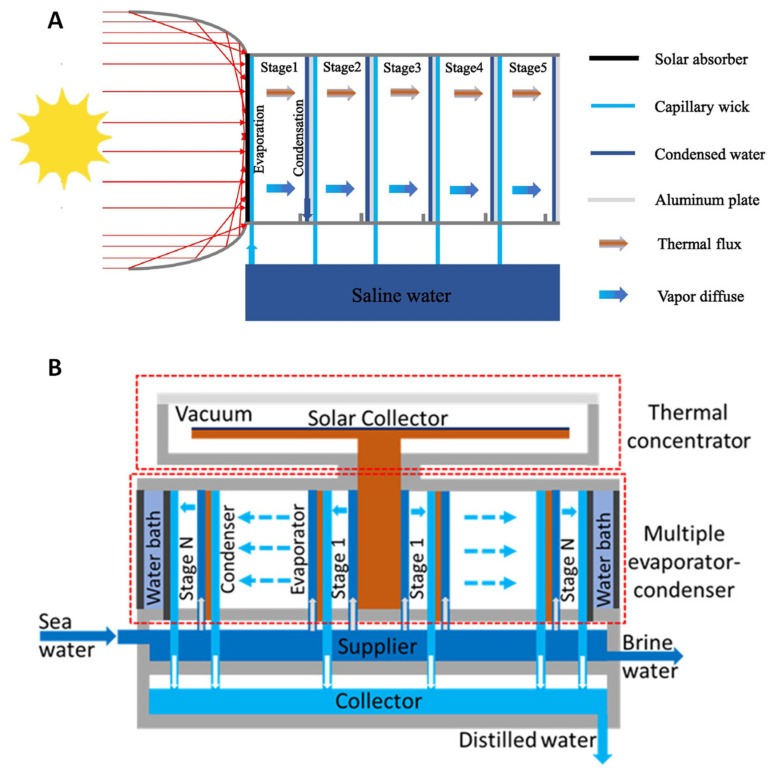
(**A**) Solar steam generation using a compound parabolic concentrator [24]; (**B**) diagram of thermal concentrated multistage distiller [25].

High solar light absorption for conversion to heat is a major requirement for SWE to have efficiency and effectiveness in clean water production. Using photothermal material absorbers alone may not efficiently absorb high amounts of solar energy, thus concentrating the light on the absorber may help in this process. This is the motivation of Li et al. [24], who developed a solar steam generator (SSG) implementing a compound parabolic concentrator (CPC) for solar light concentration (Figure 4A). The CPC is a contraption attached to the front of the solar absorber for harvesting solar intensity. This improves the overall performance of the system. However, it is critical to consider the economic factors from this additional mechanism—and this is thoroughly examined by Li et al. [24] The water flux per dollar (kg m^−2^h^−1^$^−1^) over a yearly period has shown the addition of CPC has provided slightly better results economically. The device is a 5-stage module constructed with 6 machined milled polymethyl methacrylate (PMMa) frames with a matte-black coated aluminum plate for the solar absorber. The surface area for solar absorption is 7.8 × 7.8 cm covered by a 2 mm thick glass plate for heat retention. Aluminum plates were also used for the condensation layer and were separated by a stage gap (air gap between layers) which was determined through analyzing 3 different experimental cases. A low-cost napkin was used as the capillary wick. Their study investigated 3 different cases with cases 1 and 2 tested with/without a glass cover and a comparison of 5 mm and 10 mm stage gaps. Case 3 involved an additional stage testing the significance of the improvement in performance. Case 1 displayed the lowest performance of 2.23 kg m^−2^h^−1^ and a GOR of 146.35% as there was no retention of latent heat as it was lost through radiation and convection. Case 2 displayed 2.82 kg m^−2^h^−1^ and a GOR of 185.09%, competitive with other high performing MSDs. The highest GOR was with a stage thickness of 10 mm and an air gap of 5 mm between the glass and absorber. Case 3 produced 3.5 kg m^−2^h^−1^ with a GOR of 229.21% exceeding both cases 1 and 2. Field tests have not been conducted on this device; however, a hypothetical prediction of annual freshwater production has been investigated through the modeling of annual weather in Xi’an, China. The system was estimated to produce 17.31 kg/day in a 1 m^2^ array of the device. Reviewing the economic factors during the development phase is necessary as the additional stage is considered in case 3. As discussed, additional stages result in less latent heat transfer, equating to a lower mass transfer. A continual increase in the number of stages will always provide a higher performance outcome; however, keeping the costs to a minimum is a critical factor when considering practical applications. Moreover, this should also be considered in the use of CPC as it is an additional component to the device.

In another study, Huang et al. [25] achieved solar-to-vapor conversion efficiency of 2.2 kg m^−2^h^−1^ with a 6-stage passive multistage SWE device with a thermal concentrator (Figure 4B). The GOR for the 6-stage SWE device is 125%, which achieved above the theoretical yield of a single-stage device. This design is quite different from the top-down or bottom-up approach taken by the other studies. The system is a circular-shaped multistage evaporator that evaporates in an outward trajectory, parallel to the surface level. The solar flux is absorbed by the SSA and transformed into heat—thereby thermally concentrating—then transferred to the copper plate and rod. The heat is then utilized by the system below and recycled in a multistage process. The main difference to the other designs is the direction of condensation formation and the system being in a vacuum. The heat dissipation is from the center to an outward direction where the SWE process occurs. The material mostly used is copper for the absorption layer due to its high thermal conductivity to transfer the heat to the center rod. The circular condensation layers are constructed of copper to continually recycle the latent heat throughout the multistage process. As the system is in a vacuum, the insulation layer consists of multiple layers with acrylic discs and a quartz cover attached to an O-ring. The sidewall is made from nylon material with cotton films for the wicking material. The specifics of the air gap are not mentioned in the literature and a water bath to the outmost layer is utilized as a heat sink. The limitations and challenges of this design are not outlined; however, it is stated improving the thermal concentration process can increase the overall efficiency and water production yield.

Zhao et al. [26] also presented a novel multistage floating flower-inspired solar still with high efficiency by designing a corolla-shaped concentrator and optimized through optical simulation to enhance the optical performance of the system. In this study the heat and mass transfer models were established and validated for both single-stage and multistage systems to optimize the performance. The corolla-shaped concentrator was found to have a light receiving rate greater than 0.75 at an incident angle of 20 degrees. Performance simulations showed that the water yield rate and total energy efficiency of the single-stage flower-inspired solar still were 0.85 LMH and 65%, respectively, while those of the seven-stage system were about 6.5 LMH and 480%, respectively, which is 10.66 times higher than the energy efficiency of conventional solar still systems, which are around 45%. In addition, in this study the economic analysis has been carried out and results demonstrated that the cost and payback period of the 7-stage system were 0.001$/L and 0.25 years, respectively, proving that the proposed solar desalination system with high efficiency and low cost is ideal for areas with low infrastructures [26]. 

In addition, Wang et al. [27] explored a floating multi-effect solar still that utilizes parabolic concentrators for the direct heating of the ascending seawater film. By integrating the parabolic concentrators with the still, a more streamlined and portable design is achieved. The desalination process employs a hydrophilic wick, which uses capillary action to create a rising liquid film passively. This not only simplifies operation but also minimizes heat loss. Furthermore, when subjected to intense radiation, the still generates a high temperature gradient, enhancing the driving force of vapor diffusion and ultimately leading to increased water yield. The designed floating still is composed of a front parabolic concentrator, a rear parabolic concentrator, and numerous distillation cells that are integrated within it [27]. The results of the optical performance studies indicated that the floating still has an average optical efficiency of about 80% when the light incidence angle ranges from 15 to 65 degrees. Moreover, the findings from the indoor steady-state experiment show that water productivity and GOR decrease as the light incidence angle increases, but improve with increased radiation. At an irradiation of 900 W/m^2^, the temperature difference inside the 5-effect still can reach 48.5 °C, which allows the GOR to reach over 2.2 and 2.7 kg m^−2^h^−1^, respectively. In actual weather conditions, the daily water yield exceeds up to 5 kg/m^2^/d. Overall, this study provides a promising solution to address the water demand of remote island areas or offshore platforms [27].

### 3.3. Water and Power Co-Generation Using Multistage SWE 

**Figure 5:**
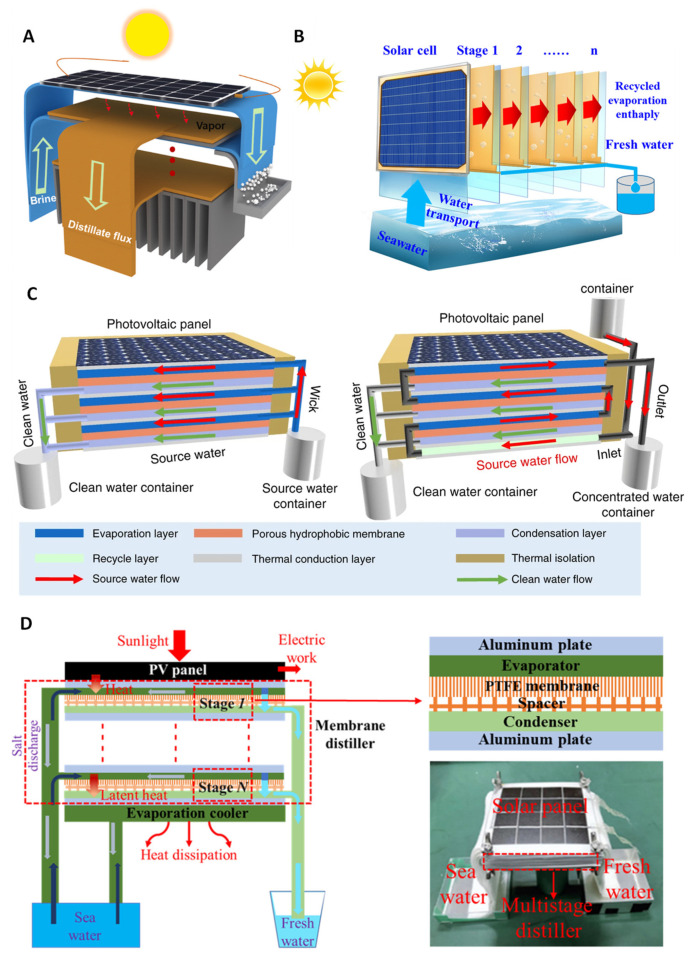
Schematic designs of (**A**) electricity, water, and salt harvesting hybrid device [28], (**B**) PV-multistage solar distillation [29], (**C**) PV-MD device [30], and (**D**) multistage distiller with PV device [31].

Water and energy are correlated with each other (water-energy nexus), thus providing a solution that can generate both clean water and energy simultaneously is an attractive approach. Recent years have seen the strategic integration of PV cells as heat source for multistage SWE, wherein sunlight is absorbed by the PV cells generating electricity, while the PV waste heat is utilized as heat source for SWE. In a recent study, Yang et al. [28] designed a PV multistage device (Figure 5A) on a passive 5-stage configuration producing 1.17 kg m^−2^h^−1^ of freshwater and an electricity output of 97 W m^−2^. The study focuses on mitigating salt accumulation in passive multistage systems as they are restricted in design with fewer water channels. The implementation of a siphon channel in the device causes the flow of saltwater through the device while the extended evaporator reduces the salt saturation within the directional flow within the system. This allows the salt to be saturated outside, perpendicular to the distillation process which can then be collected by rinsing. The device is a standard configuration of a PV multistage device using a PV panel in place of the SSA and does not comprise of an insulation layer. The capillary wick is a low-cost paper towel and the air gap in the system is 2 mm, insulated by a 1.5 cm thick polyethylene foam for heat retention. The condensation layer is an aluminum plate, and a heat sink is used in this device for residue heat dissipation. The 1.17 kg m^−2^h^−1^ was reached when operating with a 170 g/L NaCl solution with a circuit resistance of 20 Ω. 

In another recent study, Bai et al. [29] have also designed and tested a solar multistage distillation device coupled with a PV module for solar absorption (Figure 5B). It is a 7-stage distillation system producing an evaporation rate of approximately 2.26 kg m^−2^h^−1^ under 1500 Wm^−2^ of solar irradiance. The system is a top-down approach; however, it involves a tilt mechanism for optimal solar irradiance absorption. The device can alter the angle to increase the projection area of solar as well as reducing the “reverse air buoyancy of steam flow” causing a decrease in the “resistance of the steam condensation process”, ultimately leading to a higher evaporation mass. The materials used in this design are quite like the systems examined above. However, adjustments and design optimization have been added to the layers to improve efficiency. The PV module is a monocrystalline silicon cell. The device is composed of a copper plate for the condensation layer which has been chosen for its latent heat transfers across the layers. The copper plate has been designed as a “semicircular sink” for effective condensed water drainage and is coated with a “fluorine-containing nano-coating” to prevent seawater corrosion. The experimented airgap is 4 mm using a low-cost paper towel for the capillary wick. A thermal insulation of 40 mm has been implemented but does not specify the material being used. They tested the device in an outdoor environment with an average ambient temperature of 28 °C (0.85 sun). The devices have produced an evaporation rate of up to 2.2 kg m^−2^h^−1^ and an average rate of 2.06 kg m^−2^h^−1^, competitive with other devices operating under optimal conditions. 

Wang et al. [30] designed a passive multistage MD device utilizing PV cells (Figure 5C). This design possesses innate similarities with a standard SWE design but uses a membrane for an air gap. The benefit of MD is its ability to retain more heat than an air gap as heat is lost through side walls. Wang et al. [30] have stated, “heat loss from side faces is negligible” highlighting the efficiency in heat transfer through the stages of the device. The PV-MD has managed to produce >1.64 kg m^−2^h^−1^ with 3 stages under different configurations for salt accumulation management. In this study, a commercial polycrystalline silicon solar cell was used as the PV layer and a hydrophilic quartz glass fibrous (QGF) membrane was used for the evaporation chamber. The water absorbed was then condensed onto an aluminum nitride plate through an electro-spun porous polystyrene membrane. The experiments conducted in this study have produced freshwater production rates relative to the amount of electricity produced and the design of the device to mitigate salt accumulation. The investigated design has been separated into “dead-end” and “crossflow” modes where the end location of concentrated salt water distinguishes the devices. The dead-end device produced higher freshwater production, but its functionality is limited as cleaning is required from the saturation of salt-concentrated water in the evaporation layer. The crossflow design allows for a constant flow of concentrated water out of the device through a mechanical or passive mechanism to prevent salt accumulation. The difference in freshwater production is a result of outflowing water in the crossflow design as some heat is taken out, reducing the heat retention within the system. These results have produced ranges between 1.65 and 1.82 kg m^−2^h^−1^ under 1 sun illumination. 

In another study, Huang et al. [31] investigated the general co-production of electricity and water with a 4-stage device (Figure 5D). The hybrid PV-MD achieved production of 1.11 kg m^−2^h^−1^ of freshwater and 66.6 W m^−2^ of electricity under 1 sun (1000 W m^−2^). The design of this device is unique as it utilizes a 0.12 mm thick polytetrafluoroethylene (PTFE) membrane and a plastic net as a spacer in place of an air gap. This layer is fit between 2 cotton layers used for the evaporator and condensation layer. The freshwater is condensed onto a thin aluminum plate which is also used for latent heat transfer within the stages of the device. The quality of freshwater production after 2 h of desalination caused the salinities to significantly drop, and thus, require frequent cleaning of the PTFE membrane and the cotton layers. Moreover, it is stated that the optimization process is necessary as the water production of the device can be improved by using a membrane with larger porosity as it would increase the vapor flux. However, this would reduce the heat conduction, which will ultimately reduce the latent heat available for transfer between the stages. For this investigation, Huang et al. [31] used a membrane with a porosity of 0.85. 

The recent studies demonstrated that implementation of the conventional photovoltaic desalination cogeneration system on a small scale, particularly in remote and uninhabited areas like islands and reefs, is still challenging due to its intricate design, extensive floor area, and expensive investment cost, making it less economically viable [32,33,34,35]. In a study, Lv et al. developed a mathematical model and a small-scale experimental prototype to assess and scrutinize the efficiency of photovoltaic interface desalination systems. The analysis considered various factors, such as the absorption rate of batteries, ambient humidity, and other parameters to determine their impact on the water and electricity production performance of the system. According to the experimental findings, the PV interface desalination device, which occupied less than 95 square centimeters, exhibited an electrical efficiency that is 1.8% higher compared to a single PV cell, with an evaporation efficiency of more than 61% when exposed to solar irradiation of 1000 W/m². The economic analysis of the entire system indicated that PV interfacial desalination system has a lower material cost (about $1.05/m²) than the same type of interfacial evaporation material. The system is also deemed safe to operate during production and environmentally friendly to human beings [32].

Studies have indicated that a rise in a PV cell temperature can lead to a decrease in its open circuit voltage, fill factor, and power outputs. A 10 °C temperature increase doubles the aging rate of the solar panel. Hence, the implementation of effective cooling mechanisms to lower the solar cell’s temperature is crucial in advancing global renewable energy initiatives [36,37,38]. Accordingly, in a study, Wang et al. have designed a PME system featuring a passive multistage membrane distillation (MSMD) component, which is positioned at the rear of the solar cell, leveraging the waste heat generated by the cell to facilitate water evaporation. The MSMD design enables the recovery and recycling of the latent heat produced during vapor condensation in each distillation stage, which is subsequently used to drive water evaporation in the succeeding stage [39]. In this study, a 5-stage photovoltaics-membrane distillation-evaporative crystallizer (PME) was used to conduct the experiments that demonstrated a consistent and elevated freshwater production rate of 2.45 kgm^−^²h^−1^ and a reduction in solar cell temperature from 62 °C to 47 °C under 1 sun irradiation. This reduction in temperature resulted in an 8% increase in electricity production for the solar cell. Furthermore, the concentrated brine generated during the process was completely evaporated by the underlying evaporative crystallizer, thus achieving a zero-liquid discharge [39].

Another challenge encountered with the solar water evaporation system is their visual disturbance. In a study, Antonetto et al. [40] tackled the issue of environmental and visual implications associated with the installation of PV systems. As a solution, they introduce and evaluate the effectiveness of a PV module that features an innovative aesthetic surface coating capable of minimizing its impact on the surrounding landscape. The resulting compact unit was highly suitable for floating installations. The proposed solution entails some key components including an innovative aesthetic surface coating that minimizes the visual impact on the surrounding landscape, a mosaic-like arrangement of small-sized modular and passive distillers that simplifies the scalability of the capillary-driven distillation processes, and improved electricity generation efficiency facilitated by the distillation device positioned beneath the PV cell, which helps to cool it down. Under realistic laboratory conditions, it was found that the desalination rate can reach up to 2 LMH. The energy required for the desalination system was measured at approximately 670 kWhm^−3^, which was competitive with other solar desalination techniques. Furthermore, the integration of the desalination and PV modules showed improved electrical performance under optimal conditions, mainly due to the lower operating temperature of the PV module. Specifically, a relative PV efficiency gain of 4.50% was observed when the temperature of the PV cells was lowered by 9 °C [40].

### 3.4. Designing Multistage SWE for Salt Resistance or Salt Accumulation 

**Figure 6:**
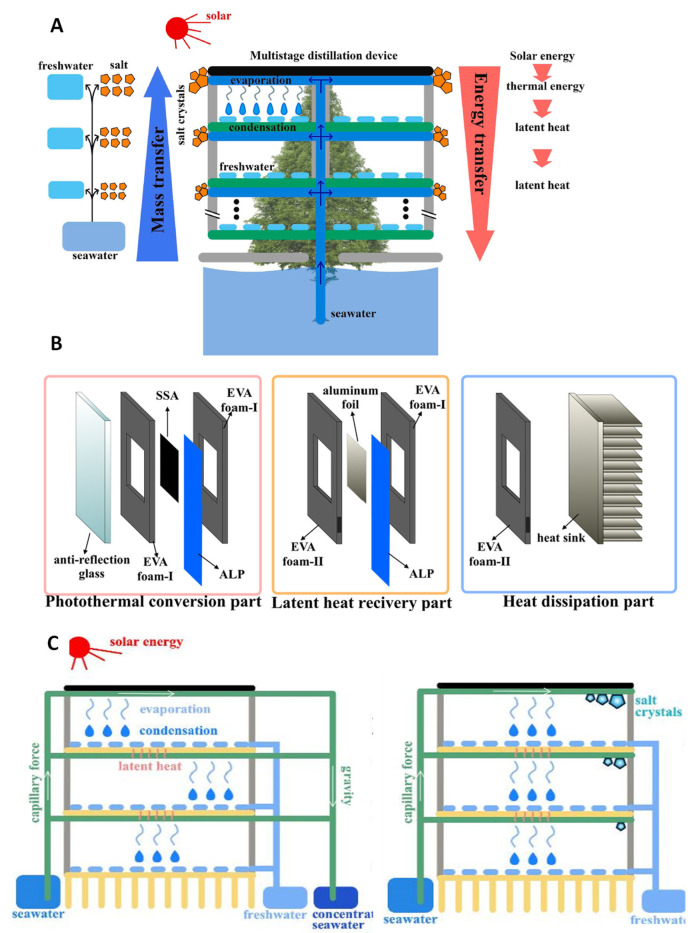
(**A**) Tree inspired diagram of passive multistage distillation device [41], (**B**) composition of the processes in the passive multistage distillation device [42], (**C**) schematic diagram of passive multistage distillation device [42].

Li et al. [41] have developed a unique design of a MSD through adopting a “tree inspired bionic structure” (Figure 6A). The performance under laboratory conditions of 1 kW m^−2^ of solar intensity and 25 °C in ambient temperature produced 2.21 kg m^−2^h^−1^. The design involves a top-down approach and utilizes a unique water transferring mechanism for the different stages. The water transport trunk (WTC) is the main mechanism allowing this to occur, located in the center of the device. The WTC operates by absorbing the seawater from the bottom and transfers in an upward direction through multiple connections of air laid paper. The materials used are SSA covered by a 2 mm quartz glass for heat retention within the system. The WTC acting as the capillary wick is an air laid paper which is used in curls along the “trunk” and flat in stages 2 to 6. The device is held together by 4 mm 3D-printed resin frames with a heat sink in stage 7 for residue heat dissipation. Aluminum foil is used as both a condenser and for latent heat transfer in the device. As the system is circular, experiments have been conducted to determine the optimal radius for the water supply rate of the evaporation layer. The optimal radius was determined to be 25 mm as data collected by Li et al. [41] showed increasing the evaporation layer area causes the water supply rate to decrease; however, it also increases the surface area for solar absorption. Long-term tests have been conducted on the MSD, resulting in an estimated 1.5 kg m^−2^h^−1^ freshwater production over a 10-day period displaying a consistent and stable performance. Moreover, field tests have been conducted under approximately 0.5 kW m^−2^ over a 10 h period and produced an estimated 10.2 L m^−2^. The freshwater produced complied with the WHO standards of 99% desalination. 

Another study was conducted by Cheng et al. [42] on a passive multistage device by replacing the air gap with an ethylene vinyl acetate copolymer (EVA) foam (Figure 6B,C). The device produced 1.98 kg m^−2^h^−1^ under 1 sun in a 4-stage configuration with a standard multistage system using an SSA and anti-reflection glass as an insulating layer. The EVA foam used in the system is categorized into two types, EVA foam I and II. Although they are both designed to improve insulation effects in place of the air gap to retain heat, the dimensions of EVA foam I and II are different as they serve different purposes for the functioning of the device. EVA foam I is 2 mm thick and is located between the insulating layer and SSA, with the evaporator and aluminum foil condensation layer acting as the main insulation for the system. The EVA foam II is 2.2 mm thick and located between EVA foam I and the aluminum foil condensation layer as it is used to release the freshwater produced in the device. The evaporator is a layer of air-laid paper with a 0.1 mm thick aluminum foil attached to collect condensation and recover latent heat. The device is unique in its use of cumulative layers of EVA foam rather than free flow of vapor between the stages.

Morciano et al. [43] have developed a passive modular solar distiller focusing on salt rejection utilizing the Marangoni effect. The device is a 3-stage system producing roughly 1.904 L m^−2^h^−1^ under 950 W m^−2^. During an 8 h testing of the device, no observable salt accumulation and decline in evaporation rate was reported. They have proposed that the Marangoni effect may be the cause of this phenomenon. The designed device involves a standard multistage configuration with an extra hydrophilic layer lined on an aluminum condensation plate. The materials used for this device are a black aerosol coated solar absorber attached to the hydrophilic evaporator. The evaporator and condenser are separated by an airgap formed by a porous plastic frame of 1.65 mm, which has been determined through experiments to prevent contamination. Both the evaporator and condenser are composed of a synthetic microfiber. To promote latent heat dissipation, a heat sink has been attached to the bottom of the device. The mechanism of the Marangoni effect takes place in the capillary wick where the gradation of salt concentration occurs due to “variations in solute concentration, surfactant concentration, or temperature along the interface” [43]. The main changes occurring in the surface tension are caused by the concentration of salt, known as the “soluto-capillary effect”, and temperature, known as the “thermos-capillary effect”. These effects can occur simultaneously, resulting in an interfacial flow towards regions with higher surface tension. This has been experimentally represented through collecting a salt sample, wherein 75% of salt concentration was removed 2 h into the experiment. 

### 3.5. Utilizing Multistage SWE for Direct Irrigation in a Hydroponic System 

One of the emerging applications for solar-driven water evaporation (SWE) is in the field of agriculture, particularly in cultivating crops through a hydroponic system. Such demand is driven by the need of clean water for irrigation purposes for different crops. Wang et al. [44] developed a solar-driven multistage distillation setup utilizing seawater to produce freshwater to feed crops in a hydroponic system. This solar hydroponic planting (SHP) system is composed of a Fresnel lens concentrator, desalination units (i.e., composed of evaporator and condenser), a secondary reflector, absorber, seawater channel, freshwater channel, and implant cavity (i.e., space for contact of the produced freshwater into the crop’s roots). The bottom part of the SHP is the seawater supply that feeds the desalination units. Sunlight strikes on top of the Fresnel lens concentrator and passes through the transparent plate. The transmitted light is then reflected into the absorber and heats up the evaporator. The evaporator of the desalination unit is a hydrophilic layer that absorbs the seawater feed and diffuses the vapor into the condenser portion of the unit, driven by temperature and concentration differences between the two layers. As water vapor condenses, latent heat is released to the next desalination unit or stage. Such process goes on until the last desalination stage of the SHP, where heat is released into the ambient environment. The freshwater distillate from the condenser of each stage is collected and channeled into the implant cavity to be used by the crop.

**Table 1:** Comparison of existing designs of multistage SWE devices and their performance.

*Item/Ref*	[9]	[10]	[21]	[25]	[30]	[28]	[42]	[31]	[22]	[29]	[41]	[24]	[23]	[26]	[27]	[32]	[39]	[40]	[43]	[44]
*Water Production Rate (L* m^−2^h^−1^*)*	5.78	~3	~1.33	2.2	1.65	1.17	1.98	1.11	1.02	2.26	2.21	3.5	4.12	6.5	2.7	0.94	2.45	2	1.904	3.98
*Number of stages*	10	10	3	6	3	5	4	4	2	7	7	6	10	7	5	1	5	3	3	10
*Energy consumption (L/kWh)*	10	3	6	3	5	4	4	2	7	7	6	10	10	7.2	3	0.94	2.45	2	2	3.98
**Multistage SWE parts assembly**	**Materials used**																			
*Insulating layer*	Silica aerogel monolith	Transparent linear LLDPE, aluminum sheet		Acrylic discs, O-ring, and quartz cover	Aluminum nitride plate		Anti-reflection glass				Quartz glass	Glass plate	EVA, covered by an AR glass		PMMA		PMMA			
*Solar absorber*	Commercially available SSA covered by AR coated glass	Spectrally selective solar absorber (TiNOX)	Aluminum plate in black coating (matt polyurethane paint)	CrAIO-based SSA attached to a copper plate and a rod	Polycrystalline silicon solar cell (Sharp) or SSA	PV panel	Selective solar absorber	Crystalline silicon solar cells	Commercially available SSA consists of cermet coated aluminum alloy substrate	monocrystalline silicon cell	SSA	matte-black coated aluminum plate		Corolla shaped concentrator	PMMA pasted reflective aluminum film, coated aluminum plate	Photovoltaic cell	Solar cell	4-cells mono-crystalline silicon solar module	Aerosol based black coating,	
*Evaporation layer, capillary wick*	Cellulose fiber (paper towel)	Hydrophilic microfiber (not specified)	PET fiber	Cotton film	Commercial hydrophilic QGF membrane	Low-cost paper towel	Air laid paper	Hydrophilic cotton	PVA sponge	Low-cost paper towel	Air laid paper (used as curls)	Low-cost napkin	Wool pulp paper	Fabric wick	Cotton fiber	Silica gel, carbonized non-woven fabric	Porous non-woven fabric	Synthetic microfiber cloth	Viscous fiber-based wick	
*Air gap, evaporating chamber*	Air gap (5 mm)	Air gap or hydrophobic microporous membrane	Air gap (9.35 mm)	Air gap	Electro spun porous polystyrene	Air gap (2 mm)	Ethylene-vinyl acetate copolymer foam I (2 mm)	PTFE membrane (0.12 mm) with plastic net (1 mm)	Air gap	Air gap (4 mm)	Air gap	Air gap	Ethylene-vinyl acetate copolymer		Air gap		PTFE membrane	Microporous polytetrafluoroethylene membrane	Air gap made by plexiglass spacer	
*Condensation layer*	Teflon AF (1 μm)—coated aluminum	Hydrophilic layer (not specified) onto an aluminum plate	Aluminum plate	Copper tubes	Commercial hydrophilic QGF membrane	Aluminum plate	Aluminum foil		Hydrophobic membrane PVDF-HFP nano fibres network on backside of PVA) onto a collector	Copper plate, coated with fluorine-containing nano-coating	Aluminum foil	Aluminum plates		Aluminum plate	Aluminum plate		Stainless steel mesh	Synthetic microfiber cloth, Aluminum sheet	2 Aluminum plates supporting hydrophilic layer	
*Frame, side walls*	3D printed nylon (Nylon PA12), Super Tuff—R	3D printed acrylonitrile butadiene styrene	Acrylic plate	Nylon materials		Polyethylene foam		Aluminum plate	Polystyrene (PS) foam	Thermal insulation layer (material not specified)	3D printed resin	Machine milled PMMa			Foam pearl cotton (10 mm)		Polyurethane foam (can be used)	Plastic films		
*Heat sink*		Aluminum		Water bath		Material unknown	Ethylene-vinyl acetate copolymer foam II and heat sink	Hydrophilic cotton layer			Material unknown							Aluminum	Finned heat sink	

AR: anti-reflecting; EVA: Ethylene-vinyl acetate copolymer; LDPE: linear low-density polyethylene; PET: polyester; PMMa: polymethyl methacrylate; PVA: Polyvinyl alcohol; QGF: quartz glass fibrous; PVDF-HFP: Poly (vinylidene fluoride-co-hexafluoropropylene; PTFE: Polytetrafluoroethylene.

Thermodynamic model of the multistage SWE-SHP system was based on its different component’s energy balance. In a four-stage model unit, its radiation, temperature change of the absorber, water yield, and its hourly GOP showed a similar trend with its outdoor experimental data. Results showed that the highest radiation reached in a day was 755 W/m^2^ at noon time, with a maximum temperature value of 56.6 °C. Water yield reached 9.12 kg/m^2^ in a day, which accounts for almost half of the first stage, with GOR of around 1.4. A multistage SWE system is advantageous to SHP system as it uses the latent heat released from one stage to another, resulting to an improved water productivity and solar thermal efficiency. In a 10-stage model, for example, evaporation efficiency reached 82.2% in the first stage and 66.2% in its tenth stage. The water yield rate reached 3.98 kg/m^2^/h and a GOR of 2.64 under 1 sun. It was observed that increasing the number of desalination stages tends to flatten productivity and GOR, recommending only 4–7 stages to be installed in an SHP setup. Moreover, the 4-stage setup gave a better economic value and efficiency compared to some recent solar HDH and stills, in terms of the cost of distilled water produced per liter (0.013) and its GOR (1.41) based on Guangzhou, China’s weather conditions.

## 4. Challenges and Perspectives

The main difference between the analyzed designs is the layouts of each stage of the systems. They incorporate layers of thermal insulation, solar absorption, capillary wick or evaporator, and a condensing layer. However, the composition of each system varies due to the chronological order of these layers and the materials being used. The materials of each separate device are shown in Table 1. Through examining the different multistage SWE devices, the performance gap between them is quite significant. Although it is not quite applicable to compare the systems purely based on their water production output as they have varying objectives, vast water production differences can be observed. 

**Figure 7:**
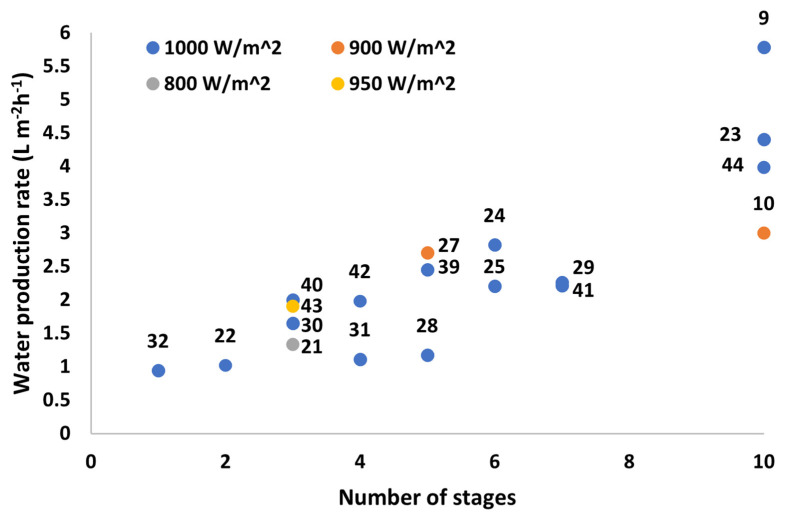
Water production rate (L m^−2^h^−1^) vs. number of stages of various multistage SWE reported in the literature (numbers in the figure indicate reference number).

Throughout the research of various devices on portable SWE desalination devices, there have been minimal tests and applications in practical environments. Systems such as TMSS and PV multistage desalination devices have collected data from outdoor environments; however, the data are limited to a single day in a specific location at a certain temperature range. In addition, in the presence of outdoor environments, the performance of the devices significantly decreases, making it an impractical solution for stable and continuous water production. As the systems are being developed to be mainly implemented in water-scarce areas with high levels of solar irradiation for low costs, the provided data are not satisfactory to provide a realistic representation of its performance. Moreover, there are currently no practical devices used to address socio-technical issues in society, and therefore, no further data aside from experimentations are available. Figure 7 shows a comparison of the water production rate in relation to the number of stages in the device and the solar intensity. It clearly indicated that increasing the number of stages generally results in a higher water production rate. However, one may note as well that the module design ultimately determines the water production rate and the overall efficiency of the system. Devices such as [10] are an outlier in this graph as it is one of the initial investigations into PV-MDs resulting in less material and heat recovery optimizations. Moreover, it is crucial to consider the materials used as the device’s ability to maintain latent heat recovery impacts the water production rate. Other areas to consider involve the duration taken for each device to reach peak water production rate. Increasing the number of stages results in a need for higher latent heat recovery and slower heat transfer across the stages; moreover, these optimal water production rates may not be reached in an adequate duration of time. This is further examined in Section 4.3.

### 4.1. Limitation of Solar Absorbing Surface Area/Wicking Ability of Materials 

One of the main bottlenecks of solar absorption capability is mostly due to the inability to continually increase the surface area of the solar absorbers. This is due to the limitation of the capillary source as there is an inversely proportional relationship between permeability and capillary pressure. The capillary pressure can be increased through the reduction of the pore size of the material; however, this will reduce the flow rate due to decreased permeability [45]. In addition, in situations where mass flow rate is lower than the evaporation rate, a dry-out phenomenon can occur, resulting in a significant reduction in the efficiency of the device [45]. Thus, an optimization process is necessary to improve the overall flow rate, while maintaining a large surface area for increased solar absorbance. 

This limitation is prominently hand in hand with the restriction of developing a device in per m^2^ structure. Currently, all reviewed devices have been small scale models which have represented a potential water production rate in m^2^. The upscaling issue is a significant limitation in practical implementation as smaller scale devices do not produce enough water. A suggestion for this issue is a configuration of multiple smaller scale devices in a m^2^ area. The wicking ability in the capillary wick and the evaporator restrains the surface area of the absorber, thus the outcome is the reduced absorption of solar flux [9,46]. Moreover, condensation can only occur when the device obtains higher heat than the ambient temperature, and therefore, the efficiency of the number of stages is also limited. When higher heat is produced by the absorber, the efficiency and water production levels increase as the growth of heat production can be utilized by latent heat recycling. Although there is a gap in the literature on the overall efficiency of surface area change relative to the efficiency increase, this is something to consider regarding the cost of production and materials. 

Meo and Morciano [45] have investigated capillary-fed vapor generators by modeling the geometrical limitations in a “reverse U-shaped symmetric configuration”. However, while this setup is not an accurate representation of all passive SWE multistage devices, it translates to many wicking processes within these systems. The research involved breaking down the capillary wick into “L section” and “horizontal evaporator” for simplicity. Specifically, for SWE, the vertical distance from the water level to the end of the horizontal section, was set to <20 mm to reduce the wicking time. The modeling resulted in a total horizontal length of 4 m, which far exceeds the length required in an m^2^ configuration. Specific to multistage devices, they [45] stated that the number of stages should meet: H > N i, where H is the distance between the water level and edge of the horizontal evaporator, N is the number of stages and i is the encumbrance of the single stage.

Leading on from this, another reason for this limitation is due to the multistage design of the devices. The current development and designs of passive multistage SWE devices follow a linear flow of latent heat, either in a top-down, down-up, or horizontal direction. Consequently, this design limits the total Q_sun_ being absorbed to the size of the top surface area. This promotes the significance of improving thermal insulation as it is the main factor in recycling the latent heat in the system. As of now, most of the designs above have implemented a thermal insulation system within the device to reduce heat loss. However, with high-performing materials, the cost is also bound to increase. 

### 4.2. Material Cost 

The composition of the system is dependent on the materials used and additional of further layers to prevent heat loss which is directly correlated to the volume of water output. This ultimately results in the consideration of the costs relative to the output of the system. Although devices such as TMSS have achieved high levels of efficiency, the material costs result in the produced water being more expensive compared to other accessible water sources in society. Furthermore, as these systems are expected to be implemented in developing and rural regions where freshwater scarcity is present, the cost of the devices is a sensitive factor to consider during development. With the technology currently available, the low-cost factor has not been met. It is also important to consider the effects of applying cheap materials to the systems as global pollution is a key factor. Using materials such as PET [47] will lower the cost of the systems, however, has the potential to contribute to global plastic pollution, especially in marine ecosystems. 

Although material costs for device construction are most apparent, the multistage SWE devices will require maintenance, cleaning, and replacement of layers. As the outlined systems are passive devices, they will not require any external energy sources aside from solar power. The cost of produced water can be determined through estimating the operating lifetime of the devices. Some research examined above has considered this as an economical factor for practical implementation. 

Other cost factors involve upscaling costs for the designs of the devices as the water production rate has been determined per m^2^ area. Not only does upscaling increase the costs for maintenance, but the costs to manufacture materials in larger sizes may not be linear. Economic analysis of these devices has not been done for most of the discussed devices as they are still in their developing stage. Upscaling and maintenance costs are difficult to estimate and value as these systems have not been exposed to environmental factors, operated for long periods and handled by different users. These factors contribute to the operational duration of multistage SWE devices as they can require careful handling and appropriate setup with current technology. Some of the literature has analyzed and estimated the costs of materials and water production cost. Li et al. [24] have done an in-depth cost investigation based on annual freshwater production over a 20-year period. The water production cost per m^3^ was determined to be USD 7.03 whereas annual freshwater production was estimated to be 6321.29 kg. Another analysis by Morciano et al. [43] displayed a cost estimation of USD 26 per m^3^, which is a much higher value. This portrays the fluctuations for costs in differing designs as materials such as heat sink, photovoltaic cells, concentrators, and mechanisms for salt rejection varies from device to device. Thus, comparing the costs between these systems may not be an adequate method to indicate cost effectiveness at this stage.

### 4.3. Reaching a Thermal Steady State

Reaching a thermally steady state has always been a challenge for passive multistage desalination devices, as their main source of heat is only from the first stage in the system. The gain output ratio is correlated to the number of stages and, as stated above, the efficiency of multistage SWE devices is only beneficial up to approximately the fifteenth stage [18]. Naturally, the heat is utilized during the vaporization process, heat loss occurs according to the insulation of the device and during heat transfer, the latent heat decreases as the number of stages progresses. The thickness of the device is reported to be correlated to the total duration for the device to reach its thermally steady state [18]. This introduces the factor of thickness being a limitation to the device’s efficiency as there can be cases where systems do not reach their thermally steady state during their operating duration. In addition, the accurate water production rate is difficult to predict and expect in practical environments as these simulations are often recorded only during their steady-state operation and “fixed value of insolation” [48]. Although the studies examined above have been tested outdoors, it is a challenge to maintain results achieved in a controlled environment in a laboratory or replicate an environment where these devices are expected to perform. From a survey of the literature [9,10,21,25], the maximum outdoor solar intensity did not reach 1 sun illumination or 1000 Wm^−2^. Moreover, the fluctuations in the intensities were quite significant with the TMSS experiencing a range from 200 to 800 Wm^−2^.

## 5. Conclusions

This review has examined the recent progression of solar-driven thermally localized multistage water distillation associated with the ongoing water shortages around regions with saltwater and accessible sunlight. The focus has been on systems with back-side mechanisms for efficient and effective multistage compositions due to their potential mainly in freshwater production. In addition, this paper has highlighted the potential for multiuse of these multistage devices as a result of their utilization of PV cells for electricity generation. However, the focus is ultimately on the overall productivity of these devices and their ability to produce freshwater under practical environments whilst maintaining regulatory standards for daily uses and low costs. With the current development of passive SWE devices, the focus on water production is not a major concern when discussing the theoretical potential and laboratory experimentation. These devices have been able to produce above-satisfactory performance under controlled conditions, with devices like TMSS achieving water production almost to its theoretical yield. However, much more study is to be completed for practical implementation as uncontrolled weather conditions significantly alter the performance of these state-of-the-art systems. The implementation of solar concentration into these devices may provide an opportunity to maintain high intensities even during cloudy and altered weather conditions. This can assist in the devices reaching a thermally steady state in a quicker duration.

We have individually discussed the various challenges and limitations reported in the literature. Although some studies do not outline the difficulties and limitations in their designs, there seems to be a common ground when looking at the performances and functions of the devices. It is concluded—as of the current technologies available—that freshwater production is limited to the total solar absorbing and wicking area. In addition, for enough temperature difference in the layers for condensation to form, reaching a steady state in the multistage configurations is another limiting factor, directly reliant on the performances of the solar absorber, heat transfer rate, and insulation of the device. Some areas to investigate are the electricity output relative to the latent heat transfer. As achieving PV cooling and freshwater production complements electricity production, this can be an ideal system for practical uses in developing regions. Moreover, limited research has been conducted on the use of membranes for heat retention in multistage SWE devices. Although the porosity of membrane systems determines the flow of vapor for overall efficiency in freshwater production, the use of membranes can allow for cleaner and improved latent heat recovery in multistage SWE systems. The materials that can be used are limited as the membrane requires high thermal conductivity whilst having hydrophilic characteristics.

## Data Availability

Data available on request.
